# Neurology

**Published:** 1904-12-03

**Authors:** 


### Progress in Medicine and Surgery.

### NEUROLOGY.

(*Concluded from page 159.*)

**Cerebro-spinal Meningitis.**—The epidemic of cerebro-spinal meningitis in New York last winter has been the occasion of several papers on the subject, some dealing with the pathology, others with the treatment. Fisher[6] states that the infective agent, the meningo-coccus intracellularis, can usually be found in the opalescent spinal fluid withdrawn by lumbar puncture during life. Too much stress cannot be laid on this in making a diagnosis. The diplococcus is found within the cells, chiefly in the polynuclear leucocytes. The onset is sudden, with acute manifestations, sometimes convulsions, generally headache, vomiting, delirium, and opisthotonus. Retention of urine is frequent. The skin is hot, and frequently covered with a rash of a dark bluish colour, resembling purpuric spots. The temperature varies between 100° and 105°, the pulse varies, it may be slow or very rapid. Babinski's and Kernig's signs are frequent. The joints are frequently swollen and simulate rheumatism. Strabismus, nystagmus, and dilatation of the pupils are frequently present. The mortality ranged between 40 and 70 per cent. There are two types of the disease—a mild or abortive type, and a malignant or fulminating one. The most frequent complication is deafness. As regard treatment, $\frac{1}{30}$ gr. of morphia should be injected three or more times a day, until the child is quiet. An ice cap should be applied to the head, bromides and chloralamid given to produce sleep. Hyoscyamine is useful when active delirium is present. Koplik[7] from a large experience, is of opinion that infants and children are the most liable to be infected; thus the oldest patient was 14 years old and 77 per cent. of the patients were under four years of age. In young infants the onset was sudden with general convulsions, vomiting, and high fever, the fever being of the intermittent or remittent type and lasting over four weeks. Rigidity of the neck, general muscular irritability and incipient wasting began within a few days of the onset. Babinski's sign was present in 77 per cent. of the cases. Kernig's sign was difficult to elicit below the age of two years, but all children above that age exhibited it. The blood invariably showed leucocytic changes. The most frequent complications were otitis media, herpes labialis and purpura. Treatment consisted in lumbar puncture, which was especially indicated in vigorous patients, the puncture being repeated after five days of symptoms recurred after (temporary) relief. From 30 to 40 cubic centimetres were withdrawn at one operation, but in exceptional cases where there was much tension double that quantity was withdrawn. Hot baths were found useful for allaying rigors, and sponging was employed to lower the pyrexia. Nammick[8] has employed intraspinal injections of lysol in treating the disease. He was led to do this from the results of treatment in the Lisbon epidemic, where the mortality with hot baths and ice to the head was 60 per cent. Then simple puncture of the spinal canal was tried in 20 cases, of which 9 died; and subsequently cases were treated by lumbar puncture, aspiration of the exudate, and injection of a 1 per cent. solution of lysol; of 31 cases 13 died, while 18 recovered perfectly. Lysol was chosen, as it is said to be five times greater in antiseptic power than carbolic acid and eight times less poisonous. Nammick's experience was much less favourable, for of five cases only one recovered. Lumbar puncture was made, purulent fluid withdrawn, and, after drainage, 15 cubic centimetres of a 10 per cent. solution of lysol injected. In one case, which recovered, this procedure was repeated four times, on each occasion with a marked amelioration of the symptoms.

Retro-ocular Neuritis.—In opening a discussion on this subject at the Oxford meeting of the British Medical Association, Gunn[9] said that under the category of retro-ocular neuritis he would include all cases in which there was evidence of the optic nerve being inflamed, apart from such as were generally recognised as papillitis. The clinical characteristics of these cases were—rapid failure of vision, particularly affecting the macular area, often in one eye only, usually accompanied by pain and tenderness in the orbit. Other characters were impaired pupil reaction to light, an absence of early ophthalmoscopic changes, and a tendency to recovery. Very similar symptoms occurred in central retinal affections, but the chief distinguishing feature was the absence of pain; also there were œdema and round pale spots in the macular region and no pallor of the optic nerve. In the early stages retro-ocular neuritis may be confounded with functional amblyopia, but in the latter there is never the diminished reaction of the pupil to light so characteristic of the former. In retro-ocular neuritis the failure of vision varies greatly, from slight dimness to a temporary, almost absolute, blindness; this visual failure always takes place rapidly, in marked contrast to the length of time a papillitis might exist without the nerve function being affected and the usual gradual failure when it did occur. The visual failure in retro-ocular neuritis frequently took the form of an absolute central scotoma, surrounded by an area in which the colours green and red were recognised imperfectly. This was due to interference with the central macular fibres. Pain is present in a third of the cases, either referred to the back of the eyeball or elicited on pressing the globe backward. The etiology varied, from an analysis of 350 cases the chief causes appeared to be cellulitis or periostitis of the obit, exposure to cold, secondary to dental abscesses, sphenoidal sinus disease, syphilis, disseminated sclerosis, influenza, gout, blood disorders, and constipation.

Intracranial Syphilis.—Pritchard[10] says that in the diagnosis of intracranial syphilis, the following rule is of great service. Given a patient between the ages of 25 and 45, affected with any form of intracranial paralysis which was preceded by headaches of nocturnal onset or exacerbation associated with vertigo and with insomnia, the insomnia occurring during the first half of the night, the paralysis developing during sleep, both headache and insomnia disappearing upon the onset of the paralysis, the cause is syphilis. The age limits should not be adhered to too closely, in that both younger and older patients may occasionally be included. The average interval between the primary syphilis, and the nerve disease was seven and a half years, the shortest interval being seven months, the longest 28 years. A recurrence of the headache after paralysis has occurred, is to be interpreted as a danger signal. Vertigo is of significance only when present with the headache, it is not as a rule accompanied by tinnitus, nausea, or deafness. The younger the patient the greater the tendency to insomnia; under 50 insomnia is the rule, over 50 the rule is somnolence. Insomnia is distinctly associated with, and a forerunner of motor and sensory symptoms. Somnolence indicates an impending mental involvement, usually a dementia. The degree of insomnia is in proportion to the headache. The paralysis takes place at night, probably owing to the fact that it is due to thrombosis supervening on endarteritis, and the circulation is less rapid when asleep. When paralysis develops during the day it suggests ruptured aneurism rather than thrombosis. The syndrome applies peculiarly to cerebral syphilis, in a much less constant degree to spinal syphilis, and scarcely at all to hereditary syphilis. Persistent nocturnal headache with associated insomnia properly interpreted and properly treated should not be followed by paralysis at all. The prognosis in syphilitic disease of the central nervous system varies widely, and cannot be predicted with any certainty in advance of at least some initial response, or lack of it, to specific treatment. Meningitis, gummatous tumours, many cases of myelitis and paralyses due to thromboses offer a decidedly favourable prognosis. On the other hand locomotor ataxy is never cured. Unless syphilis is promptly recognised and treated the outlook is bad. Some cases of pseudo-paresis clear up under large doses of iodide, these cases not differing in symptomatic details from others which steadily progressed from bad to worse. In treatment mercury is indicated the nearer the invasion is to the primary disease, the longer the interval the more positive is the indication for iodides. Iodism is less likely to develop if progressively larger doses are given than when a fixed dose is steadily continued. Thus iodism occurring with a continuous daily dose of 20 grains often disappears when 40 or 50 grains are given.

**Post-diphtheritic Chronic Bulbar Paralysis.** — Harris [11] has published an account of a third case of this rare condition. As a rule all cases of diphtheritic paralysis either recover perfectly or die from the disease, but exceptionally cases occur in which the paralysis remains permanently, and the cases recorded are examples. All three cases commenced with palatal palsy and nasal regurgitation, from three to six weeks after an attack of ulcerated sore throat, which persisted from four to seven years. Two of the cases recovered, one completely after six years and the other partially after seven years, while the remaining case, after suffering for four years, is slowly becoming worse, the palatal palsy having become complete and laryngeal palsy commencing. The treatment adopted was the administration of as large doses of strychnine as the patient could bear, with the application of galvanism.

[6] Med. Rec., Aug. 13. [7] Med. News, June 4. [8] Med. Rec., June 4. [9] Lancet, Aug. 6. [10] Med. Rec., May 14. [11] Lancet, July 13.

